# Prec(ar)ious knowledge and the neoliberal academy: Towards re‐imagining epistemic justice and critical psychology

**DOI:** 10.1111/bjso.12617

**Published:** 2022-12-28

**Authors:** Michelle Fine

**Affiliations:** ^1^ The CUNY Graduate Center, Critical Psychology, The Public Science Project CUNY New York New York USA; ^2^ Visiting Scholar at University of South Africa Pretoria South Africa

**Keywords:** critical participatory research, decolonizing psychology, dispossession, precarity

## Abstract

This epilogue is written in the ink of gratitude and provocation, reflecting on the essays that constitute the special issue on precarity. I briefly review the key gifts of the essays and then try to imagine how a social psychology of precarity could be theorized and engaged *otherwise*, with commitments to epistemic justice, designed with decolonizing methodologies and organized in solidarity with movements for social justice.

## BACKGROUND

In *Imagine Otherwise*, Kandace Chuh draws on postcolonial and transnationalism studies to make visible how strategically Asian American literatures and US legal discourses are structured through normative claims about race, gender and sexuality. Chuh (2003) invites readers to imagine Asian‐American studies *otherwise*. In cross‐disciplinary synchrony, the present transnational volume of articles provokes an imaginary for a social psychology of precarity, *otherwise*. These articles, anchored in theory, disciplinary critique, transnationalism and commitments to decolonizing, carve new paths for theorizing, organizing and studying what the editors have called an ‘unbounded understanding of precarity’ (see also the works of Tina Campt; Decolonial Psychology Editorial Collective).

Invited to pen an epilogue, I hope to chat with readers about what we have learned and loved in these articles and how we might move forward as we research/write about a condition barely graspable and yet corporeally so familiar; the existential, ever present, pervasive and viscously unjust condition of precarity in which we are all drowning, unevenly of course.

In this epilogue, I note with appreciation the conceptual and methodological moves these authors have advanced. I have no doubt it is difficult to write through the multiple conjunctural crises (Hall & Massey, 2010) in which we find ourselves, saturated in images of refugees washing up on shores; billionaires accumulating dollars, bitcoin, euros, pesos and rubles, colonial land grabs; children with no clean water; authoritarian regimes rising to power; racialized capitalism bleeding across empires; police and militaries assaulting and murdering with impunity; neoliberal logics insinuating and corroding public institutions; polar bears sitting awkwardly atop melting circles of ice, and women/femmes being beaten—precarity at home—across the globe. It is not easy to make sense and justice, from within the present moment—and yet we must try.

In 1929, from a prison cell in Italy, Antonio Gramsci wrote: ‘the crisis consists, precisely, in the fact that the old is dying, but the new cannot be born and in the interregnum, a great variety of morbid symptoms appear’ (as cited in Bauman, 2012, 49). A century later we are, of course, still and always, in the interregnum—the between—surrounded by morbid symptoms and experiencing precarity at multiple levels coursing through our global veins; trying to make sense and make justice.

These articles address, in part, how social psychologists have colluded in the production and (mis)representation of precarity, by obscuring the structural/colonial/historic behind the psychological. But perhaps as compelling, these articles posit our responsibilities as critical scholars to name the conditions that produce precarity and the stratified consequences that unfold. The writers offer a travelogue of precarity forms—from occupation of lands and homes in Palestine to job loss, to being mis‐raced or confusingly raced as white—a range of disturbing destabilizations of place, economics and self. At the same time, these articles gesture towards the radical possibilities for a critical psychological praxis grounded in epistemic justice, decolonizing and participatory commitments, aligned with movements for justice; social psychology, *otherwise*.

As these incredibly rich and wildly creative articles reveal, precarity is multi‐scalar; the perfect object of inquiry for social psychologists eager to theorize and track how the economy, politics, ideologies, environmental crises, racial capitalism seep under the skin, invade our homes, workplaces and our sleep, render some of us mute, some of us resigned, many of us despairing, lots of us activist and rageful and an elite few willfully ignorant.

Lewin (1951) might say precarity is an elegant social psychological construct, a dynamic not a variable‐spawned promiscuously at the person‐environment membrane.

To establish a common working definition, let us assume precarity to be constituted through systemic disinvestment in/disruptions of taken‐for‐granted opportunities and material conditions, colonial and corporate land grabs, state violence enacted in drag as ‘protection’, everyday violations of living/learning and the unnerving predictability of impending disaster. These disruptions are metabolized through racial and class hierarchies, with profoundly differential consequences for elites and those surviving in what Harney and Moten call the ‘undercommons’ (Harney & Moten, 2013). Precarity extends dispossession under the skin (Fine & Ruglis, 2008); an economy of affect and uncertainty tithed to structural loss–or the teetering possibility of loss. The pooling and coagulation of precarity‐inducing policy consequences can be found most viscerally and viciously in the swollen ankles of communities of colour, poverty and immigration, those exiled from the category of (hu)Man (Wynter, 2015).

Precarity can rupture when a factory closes, Israel annexes a community on the West Bank, a Child Protective Services worker investigates a family in Texas loving and caring for a trans child, when Global North environmental pollution devastates communities and islands in the Global South, when a neighbour is reported as an ‘accomplice to an abortion’ in Idaho she drove her (grand) daughter to the state border, the parent of an elementary school child in Florida calls the authorities because you ‘said gay’, a border agent decides to rip your child from your arms after a long and treacherous journey from Central America to the US border, you called the police for help with a mentally ill child, and they shot her, while Black. Precarity is a downstream cattle prod for humans, mobilized these days, maybe always, as a sadistic weapon of capital, empire and colonialism, landing with painful regularity along well‐trodden lines of race, ethnicity, gender, sexuality, class, caste, disability… No coincidences here. Precarity lives in our bones and, grows in triplicate in the academy—as we witness, endure and reproduce its cascading and destabilizing tremors.

Enter critical psychological studies on precarity. As evidenced throughout this volume, *conceptually* these authors obligate scholars to theorize and reveal how precarity attaches to material conditions, institutional betrayals, racialized traps, histories of colonialism and to refuse the ‘coloniality of knowledge’ that aligns with power (Mignolo, 2012). *Epistemically*, they insist that psychologists unearth mechanisms of structural *and* intimate violence; make visible the spiderwebs of complicity and resistance and healing that connect everyday lives with historic and structural conditions; collaborate always with and centre the perspectives of those most impacted (Fine & Torre, 2021); suture the psychological with the historic, the structural, the colonial (Weis & Fine, 2012) and never isolate stories of oppression from flows of unjust privilege, collective enactments of resistance and radical alternatives not yet.

## FLYING MONKEYS AND PRECARITY

**I'll offer an origin story**; the story of how I tripped into ‘precarity’ when speaking with youth attending deeply disinvested schools in 2014. Over the past 35 years I have testified in more than a dozen court as an expert witness; the focus is always educational injustice lawsuits on gender/race discrimination, finance inequity, disparate impact of testing policies, zero tolerance and educational inadequacy in communities of poverty. I am typically invited into lawsuits set to address what Rittel and Weber (1973) call *wicked problems*—entangled, crusty, reproductive, with many origins and mutations; but courts want *soluble remedies*. I enter with a basket of evidence of policy‐induced precarity, evidence gathered with/alongside/from and for young people who have paid the most severe price for an under‐resourced education system and over‐resourced carceral geographies. As a scholar in court, I smuggle in numbers and narratives to paint scenes of cumulative and cascading precarity pouring into communities of disinvestment and structural abandonment, suing for a token of justice. As an ‘expert witness’ my task is to assure the court that the aggrieved, historically oppressed and disinvested community is ‘worthy’; that they experience the outcome and are not the cause of structural inequities. My job is to challenge dominant lies, as Ignacio Martin‐Baró (1996) would argue; to flip the script as W.E.B. Du Bois did in *The Philadelphia Negro* (Du Bois, 1903a), tracing meticulously how racist policies, histories and structural arrangements pour ‘troubles’ into communities of colour, and then blame those communities for their own problems. On the stand, I trade my white ‘authority’ voice of the Ph.D. to legitimate what Du Bois called the well‐known moans of the darker race, or the Sorrow Songs (see Du Bois, 1903b, chapter 14) which he knew would only be attended to when a profit could be made. Even writing these words is repulsive.

The courtroom is a wrestling match for duelling research narratives. The dominant story enjoys well‐funded lawyers, who can mobilize lots of evidence demonstrating why these children are so damaged, beyond repair of state investment; why ‘money doesn't matter’. The counter story must be framed to chip away gently at the dominant story with legal logic, empirically demonstrating harm, need and capacity and suggesting that the prescribed remedy—always inadequate—will miraculously resolve the scalding, cumulative and deep scars of multi‐generational dispossession.

In 2014, I was asked to testify in a lawsuit arguing that poor, immigrant children of colour in California deserve more, but receive less ‘instructional time’ in public school because of finance inequities, leadership turn‐overs, lockdowns due to gun violence on the streets, interruptions, 40% long‐term subs, immigration raids, over‐reliance on testing, over‐heating of the buildings, etc. With then graduate students Cory Greene and Sonia Sanchez (Fine et al., 2016) we undertook archival analyses of public school databases, participant observations, visits to schools, focus groups and interviews, we met with groups of adolescents and educators from the plaintiff schools in San Francisco, Oakland and Los Angeles. In each city, the students—sitting in groups—individually completed a short quantitative survey about school/work life, checking off a list of ‘intrusions on time’ and ‘frequency of occurrence’. Then, with more creative and catalytic methods, participants were given blank paper and magic markers and asked to draw maps of ‘how time feels in your body in schools’. A vague, kind of weird, but openly generative prompt. We amended a cognitive/identity mapping method, deployed by Jodelet and Milgram (1976) in the 1970s when they were studying *psychological maps of Paris*. Mapping—of identities, places or experiences—as a method typically elicits creative, representational landscapes of identities, places, experience and/or affect (for history of the method, and examples, see Futch & Fine, 2013).

With no hypotheses, and truly interested in the wide landscape of possible responses, the map prompt was quite open‐ended: *What does time feel like in your body in school?*

It was in our very first focus group, when a student introduced himself as ‘I am Carlos ‐ Chicano, gay, president of the Senior class at Roosevelt High School’. He went on to narrate the picture he drew of himself and his classmates walking along ‘the yellow brick road. This is my life – I am good at school and I stay. But all along, we take tests, some of us do well and keep going, and some fall off the road. We don't even know what's behind the green curtain, if there is anything. We just keep walking’. His references were to a popular childhood film, *The Wizard of Oz*. And then he hesitated, and said to his peers, ‘But you're walking down this road, and there are these flying monkeys, you know? They keep getting in the way’. As if they understood the metaphor automatically, the other students chimed in: ‘Yeah I get what you mean about flying monkeys’—Alicia interjected, ‘My brothers are both in prison but they call me every morning to make sure I am ready to go to school, they worry about me so much’. Jeanne chimed in: ‘Not to be, like you know, pity or anything, I just lost my little brother this summer, so um, and that was something that was really hard for my family to deal with. He was loved by a lot of people. He was only like twelve, and um…it, it, it all has to do with what you're anchored in. So I just wasn't sure if I should travel to Princeton University this summer’. A few moments later, Marcello added, ‘My dad was deported last year and my life has been pretty rough since then’. Listening to precarity as narrated by young people living in the shadows, the theme of ‘flying monkeys’ became a telling metaphor for the structures and unpredictability from which their young lives dangled; a metaphor for precarity. In focus groups following the mapping, students sketched how the carceral system strangles their kin network: siblings in prison, being removed from family and placed in foster care, involvement with the juvenile justice system, participation in gangs. They spoke of life difficulties and yearned for support. Narrating stories not of damage, but *dispossession and desire*, these young people speak the wounds and the yearnings as they analyse the morbid symptoms that entangle their lives.

Asked to explain his map of time, Edward told us he was anxious to imagine tomorrow.
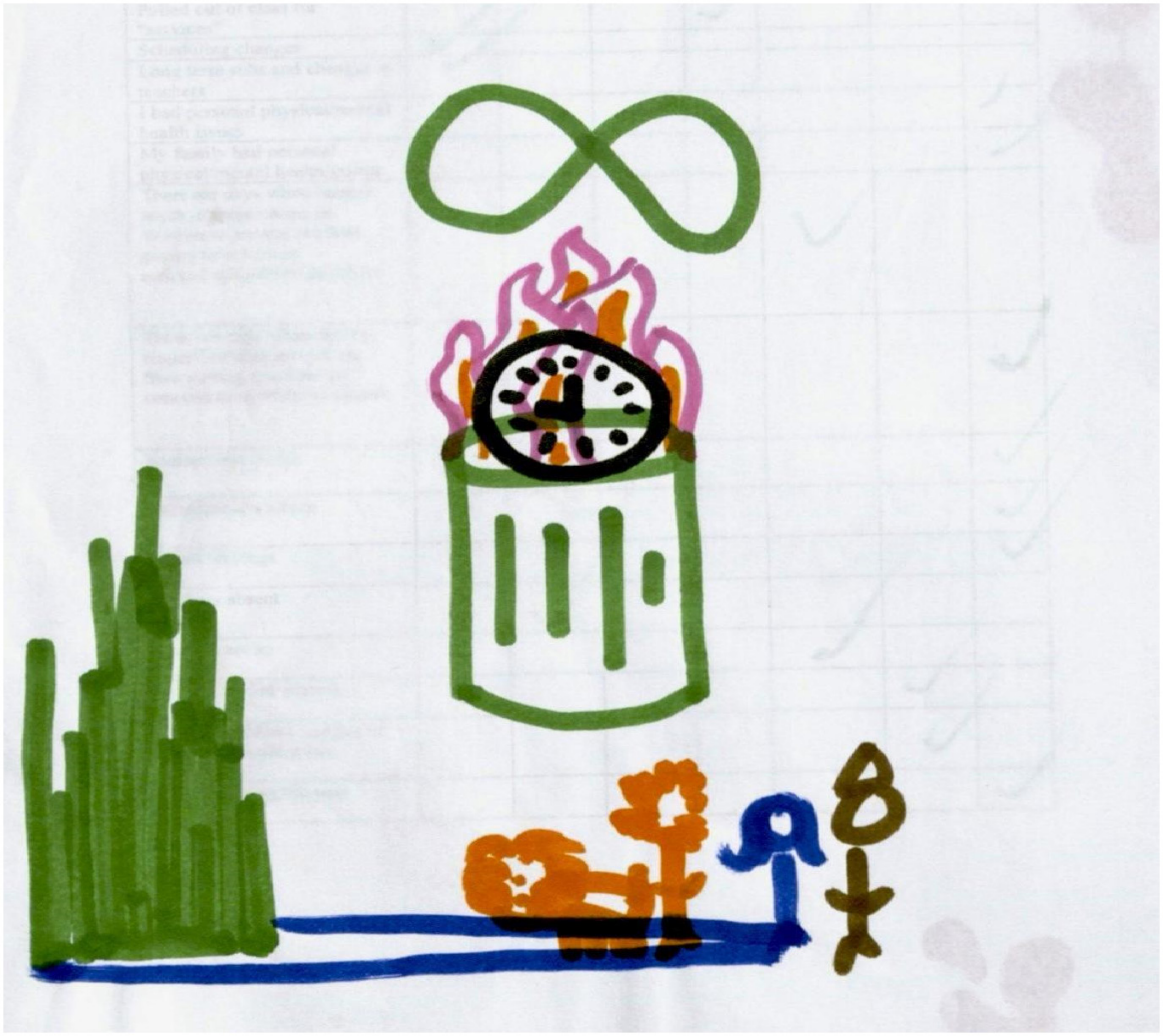
 
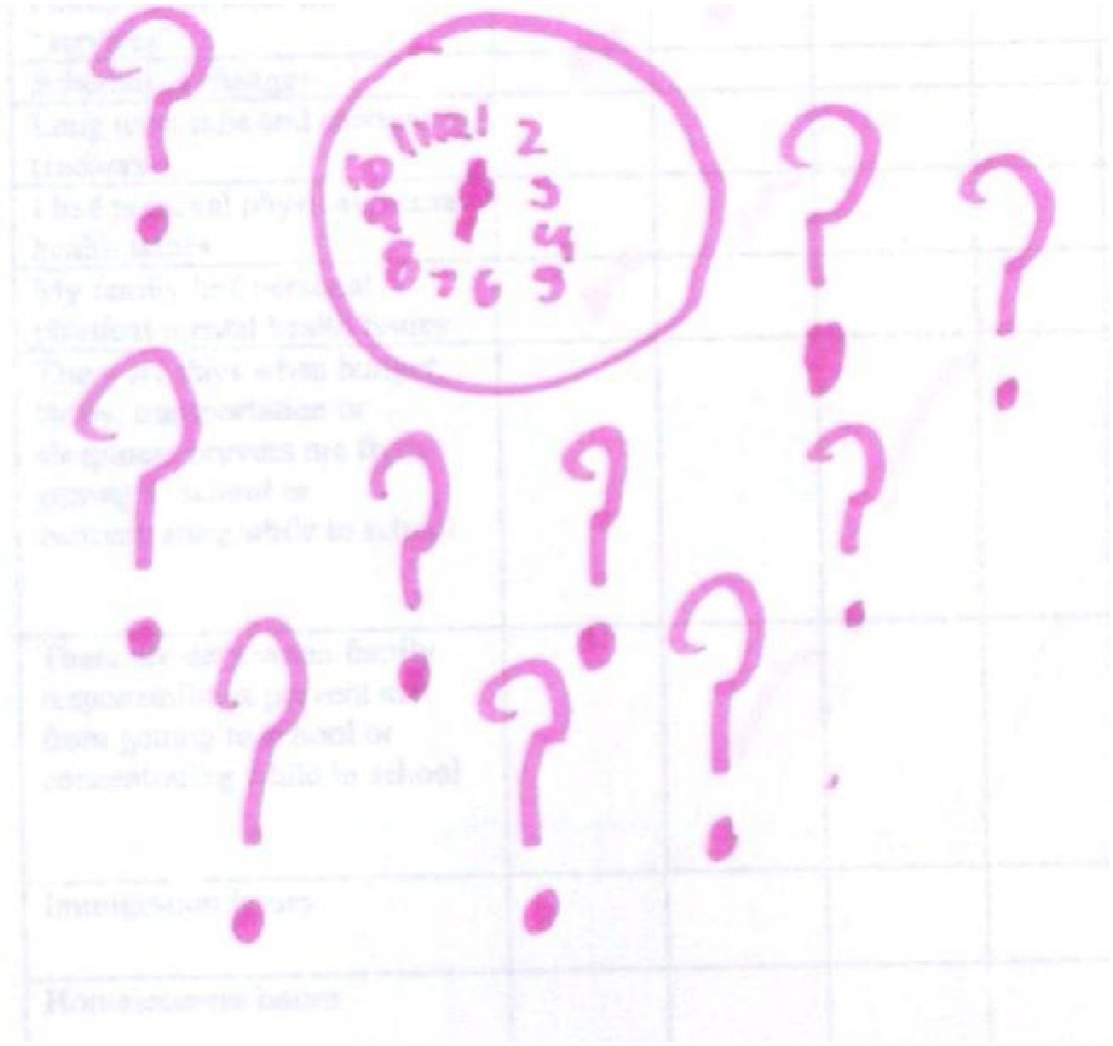
What does time feel like in school? Edward's picture

**Edward:** *But it's just a clock with a bunch of question marks, because how I think of time is, it's you never really know what to expect from it, …I do not know, I think you can never trust, you know. It's always unexpected*.

It was as if he were echoing critical theorist Isabelle Lorey, ‘Precarization means living with the unforeseeable, with contingency… The conceptual composition of “precarious” can be described in the broadest sense as insecurity and vulnerability, destabilization and endangerment’ (Lorey, 2015, p. 10).

Precarity is laser‐like in purpose but promiscuous in form; a shape shifter, eager to expand. Precarity swells, breeds more morbid symptoms and then is criminalized. In the United States, the answer to school chaos is policing. And this too was evident in the maps of the students. As you can see below, Meg makes clear that when she is in a ‘high track’ (high stream) class, top students are treated with dignity, invited to engage in inquiry and praised for their creativity—but not when she is in a ‘low track’ (low stream) class. ‘High achieving students’ are segregated from the mass student body. In ‘low track’ classes, as she sits with struggling students, we see images of children in cages, and she speaks of armed personnel in the halls.
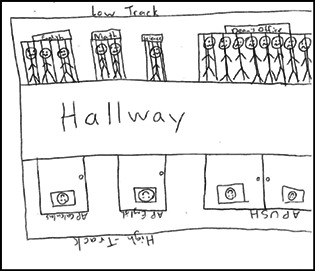
Megan's map on what does time feel like in school?

**Meg**: ‘*Big, ginormous rifles, like, on their back, just walking, “Hey, good morning.” It's normal at our school’*.

**Researcher**: *What rifles?*

**Meg**: *Like, there's a police station—yes, rifles*.

**Jalill**: ‘*Oh, I was just going to add onto the thing like talking about how our schools resemble jails… recently our principal that I guess came to the school just like two years ago or something like that, he just um brought in this new policy where they do random searches with like dogs’*.

**Chris:**
*‘It's not even random. They pick like the lower classes, and then—I swear, and they're like, everyone count of numbers, one, two, three, they keep track, who picked number three? And they're like, all the three's step outside. And the dog will search you. And it's never an AP class [Advanced Placement or “top stream” classes] or anything like that. It's always the lower—'*

**Jahill:**
*‘Our school, they randomly search your locker and then leave a note in there that just says, ‘We searched your locker’*.

Precarity cultivates suspicion of Others, which heightens surveillance which usually ends poorly for children of colour and children in poverty. Again, these *chains of suspicion* attach by design to protect privileged children and to contain the bodies of children of colour and poverty.

An embodied sense of uncontrollable and persistent change has been called ‘root shock’ by psychiatrist Fullilove (2004), who has studied the intimate psychic consequences of uprooting, disruptions of relationships and erosion of communities of meaning, particularly within communities of colour. People who have been displaced and surrounded by constant change experience ‘root shock’, a traumatic stress reaction to loss of some or all of one's emotional ecosystem. These psychological concerns unfold because communities are changing but, more importantly, because residents are betrayed and feel they have no control over the vast changes, which threaten the intimate webs of social relationships that help people endure.

While Fullilove focuses on the psychic trauma of root shock, neuroscientist Bruce Ewen has studied the neurological consequences of disruptions and oppression on children's health (see Mc Ewen & Stellar, 1993). McEwen and colleagues have conducted extensive research on *how* external stressors move under the skin, into the bloodstream and nerves of children. With a range of physiological indicators, McEwen tracks ‘allostatic load’—the ways in which external conditions penetrate bodies, including noise, lack of heat, over‐crowding, police presence, exposure to violence, affecting children's hyperactivity, hypertension, blood pressure, diabetes and other health conditions. More contemporary work on socio‐economic disadvantage and youth development, how oppression crawls into our blood and nervous systems, can be found in the writings of Raffington et al. (2021) as well as Bridget Goosby et al. (2018) who study pathways linking racial discrimination and other forms of social marginalization to racial inequities in health over the life course and across generations. Cheadle, Goosby and colleagues, in particular, combine biological markers and biosignals to study how racism gets under the skin and impacts the health and chronic disease risk of impacted groups (Cheadle et al., 2020).

While a significant sub‐line of critical neuroscience demonstrates the plasticity of these effects, how supportive environments, within family and/or schools, can mediate and reverse the impact of these stressors, for these young people in California home, school and community were impacted by environmental stressors and pervasive disruption. The combination renders these young children brutally vulnerable to stressors and the damaging consequences of cascading precarity, uninterrupted. Precarity is a deposit placed in bodies/communities/neurological systems through a theatre of state‐sponsored violence and sadism, rehearsed again and again in the realm of the social, worsened when state policies refuse to provide the most minimal of protections or buffers. Important to remember: There is a brilliance, a wisdom, borne in these conditions—as we heard from Carlos and friends. But it comes at a price. I turn now to consider the gifts of this volume.

## THE GIFTS OFFERED BY THE PRESENT VOLUME

The present collection of essays is a gift to critical psychology: theorizing and researching *precarity* as a yeasty construct, one that lives and mutates and swells and shrinks, in the membranes of people and the state, everyday persons and racial capitalism, rising white supremacies and militarized colonialisms, swelling carceral geographies. Borne in interdisciplinary theoretical soil, with samples drawn from around the globe, the arguments set forth in this volume interrogate multi‐scalar questions, relying upon a range of creative epistemic commitments and methods and powerful new frames for critical psychology. Precarity is explored here as a construct that affords social psychologists, in fact obligates us, to take seriously how economics/labour/politics/policy/ideologies insinuate under the skin, in our everyday relations and our psyches, as Fanon (1961) implored us more than half a century ago. While each article alone carves a rich space for expanding our understandings of precarity, across articles I underscore four interventions into social psychology advanced by these writers.

### Suturing personal subjectivities and the material world: economics, viruses and the melting planet

This is (unfortunately and still) a fundamental advancement in social psychology; these writers understand subjectivities as entangled with structures of class, labour, gender, sexuality, race, human (in)securities, borders and show us how to theorize and research with multi‐scalar lines of analysis. As noted by Adam‐Troian et al. 2022 precarity invites social psychologists to theorize how ‘subjective experiences of permanent insecurity’ are derivative of ‘objective material strain’. These articles caution against dismissing ‘[the] apparently irrational language of conspiracy’—as psychopathology, ignorance or evil and ask readers to consider, instead, ‘how might such marginalized forms of thinking be a consequence of social precarity?’

As a more specific interrogation of how the demands of ‘flexible capitalism’ wears on the bodies of working‐class people, the Schmitt et al. 2022 offer a quite compelling analysis of how time–space distanciation (TSD) impacts working‐class persons in varied regions of the United States. These writers examine if and how, a high TSD, as an aspect of ‘modern capitalism’ widens disparities, reduces flexibility, imposes disadvantage and layers on substantial stressors for poor and working‐class people, while parading as ‘flexibility’ and ‘choice’.

Mahendran et al. 2022, with interdisciplinary theorizing and stunningly creative methodologies, situate subjectivities within an even more expansive field of global dynamics where precarity and migration intersect with agency and desire. They explore global and planetary consciousness with two interactive methods—the IWAH (identification with all humanity) global identification scale and the IWMT (international worldview mapping tool). Interested in ‘cross‐border cooperation’ as essential to ‘climate change, refugee‐related movements and future pandemics’, they note with concern ‘low global identification scores’. From England, Scotland and Sweden, participants are positioned to ‘rule the world’ by moving or removing borders. These writers seek to understand why global crises—the pandemic, immigration and environment—stir fear and demands for heightened separations and borders for some, as others design a world with few or no borders. This stunning little paper provokes a range of intriguing questions about how socially engaged psychologists might nourish planetary consciousness. Together, these three articles intervene meaningfully into social psychology, investigating porous boundaries of Self, Others, economics, viruses and global warming, embedding ‘individuals’ within a messy global environs.

### Interrogating the academy as a neoliberal space of precarity

A few articles focus specifically on the academy, as an instance of structural precarity, making visible the neoliberal waters in which we swim. The researchers place the academy under an analytic screen, tracking neoliberal logics that saturate and corrode our universities, leeching off contingent labour and students' dreams, particularly troubling as universities circulate, enforce and legitimate exclusions and hierarchy in the pernicious name of ‘merit’.

Albayrak‐Aydemir and Gleibs 2022 theorize what they call *academic precarity* at the nexus of ‘subjective experience and as an organizational practice’, that ‘bolsters an inequality regime’ in the academy reliant on ‘precarious academic positions and practices’. They call for scholarship/policy/organizing across institutions to ‘make inequalities more visible and decrease perception of legitimacy’ (of the existing system). Rooted in the United Kingdom where 72% of full professors are male, 125 of the 19,285 professors are Black and only 35 of these Black professors are women, these writers cast a powerful shadow over the academic regime striated with neo‐liberal logics and temporary contracts, racial and gendered disparities, and self‐legitimated through the impenetrable construct of ‘merit’ as if ‘neutral’ and ‘objective’.

A number of articles in the volume trouble the academy as a space of tripled precarity blues: the university is of course *subject to* the corrosive impact of neoliberal logics; students and faculty *experience, witness and collude* in (by desire or not) the slow violence of neoliberal penetration (even as many of us protest/resist) and, third, the academy as an institution *valorises* capitalist, colonial, white supremacist and patriarchal metrics of ‘merit’ and productivity.

### From whose point of view? Challenging the uncontested colonial gaze of social psychology

While the academy is an object of scrutiny across a number of papers, many also drill down into the intellectual and political project of social psychology, a discipline rooted in Western eyes and white gaze and aligned with colonial projects and troubling categories. Turning inward onto social psychology as a neoliberal disciplinary engine within the university, Hakim et al. 2022 systematically examine a set of empirical articles focused on the ‘Israeli‐Palestinian conflict’ to make their case about how social psychology reproduces uncritically assumptions that legitimate material and epistemic injustices: the Israeli occupation and research aligned with a Zionist gaze. With careful and precise attention to the language of these social psychological studies, the authors make visible how historic and contemporary power asymmetries and occupations are obscured in these studies, hidden beneath the language of ‘intractable conflict’. They contest the ‘two side‐ism’ of these articles, (mis)representing the conflict as a battle among equals and obscuring the violent dynamics of occupation. They note that two‐thirds of the outcomes studied fail to mention material claims to land, homes, travel/mobility access, right to return for Palestinians and operationalize, instead, desired outcomes as positive emotions and attitudes between Israeli Jews and Palestinians. As they note, ‘This article considers a situation of precarity – the historical and ongoing dispossession and displacement of Palestinian people as a result of the Israeli‐ settler‐colonial project – that would seem ripe for social psychology analysis as an exemplary case of precarity’. This article unveils what we might call an *epistemology of colonial obfuscation*, when the authors ask: ‘How does colonial violence manifest in knowledge production about conflict?’ They conclude, ‘Our findings highlight how the investigation of the context can carry a colonial standpoint that reflects and serves interests of ongoing domination. A more effective social psychology of precarity in the case of Palestine or elsewhere requires great attention to such dynamics of the coloniality of knowledge’.

### Critical psychology studies of precarity: radical imaginaries within social psychology

While much of the intellectual labour in this volume addresses the problematic colonial imprint and gaze of social psychology, within most of the articles one can find a glimmer of how social psychology might be taken up *otherwise*, by engaging participatory inquiry, generating visions of a world without borders, inspiring global consciousness and reorienting ‘hegemonic social psychology with the aim to disrupt the politics of knowledge production and eradicate precarity.’ (Reddy & Amer, 2022).

In Rua et al. (2022), the writers introduce *participatory action research*, a form of epistemic justice that honours and centres the wisdom, experiences and lines of analysis held by those most systematically impacted by historic and contemporary injustice. In their work in Aotearea, New Zealand, the writers introduce the *Mãori precariat* as an assemblage, refusing to essentialize or homogenize the community: ‘the precariat encapsulates discrete groups whose lives are burdened due to the pernicious effects of having to survive on low incomes or welfare supports and to navigate various socio‐political exclusions, insecurities and discriminatory practices’. Rooted in indigenous epistemologies, the authors introduce a set of vibrant ‘cases’ of critical participatory praxis rooted in the needs, desires and commitments of community: a *Kaupapa Mãori research informed approach*, conducting research ‘towards increased socio‐economic justice and emancipation… by employing participative research strategies that centralise the voices of Mãori’; *the design and circulation of a popular book and Thinkpiece* for the government on the ‘intersecting issues of insecurity within the precariat …dispelling hegemonic myths and offering human alternatives to the penal welfare system’; research that documents the *impacts of low paid and insecure work* on Mãori, collaborating with Documentary Theatre animating the struggles induced by neoliberal government policies, bridging what they call precariat households and policymakers. This is deeply public facing, decolonial and intentional work—accountable to communities under siege with an expansive range of audiences.

Lukate 2022, in very different ways but with equally thrilling provocations, offers an important set of ideas in a self‐reflexive essay interrogating the categories that social psychology so loves to valorise, categories that do not travel well over (or even within) national borders, categories that flatten and reproduce a white gaze/Western eyes. ‘By bringing together phenomenological interpretation and [uncomfortable] reflexivity… exploring the limits of researcher and researched positionality in making sense of White as a precarious address’. She explore the ‘precariousness of everyday address categories’—erupting when one is recognized differently than one thinks of themselves; destabilizing a sense of synchronicity between how one sees self, and how Others see me. Reminiscent of Fanon's, ‘Look mama a Negro’, in this case, being seen/named as white.

In a poignant section on critical (and awkward) reflexivity, the writer reflects on ‘culture specific inner eyes’, borrowing from Sylvia Wynter (2015), asking ‘scholars to give more attention to how our inner eyes limit how we name, describe and theorise our research’. Challenging the ways in which Western, hegemonic categories overlay how researchers name our research and participants, the paper takes as an object of inquiry how four women from Germany and England make sense of being called White, Oboronyi (stranger/foreigner) or mzungu (white settlers) during travels to Africa. With pluralistic and analytic imagination, Lukate brings together an interpretive collective, to unpack the layers of uncomfortable precarity of address in these seemingly benign but meaningful (mis)interactions. She asks how can psychologists ‘advance a psychology based on an African cultural worlds view’… no longer locked in ‘western binary logics’ and refusing ‘the multiplicity of realities?’ The paper ends, provisionally, with a call to ‘disrupt the coloniality of knowledge, with a willingness and ultimately commitment to “scrutinise and crack” our ways of naming, framing and doing research’. This paper opens an aperture for how we might critically engage precarity studies across the Global North and South, with a humble willingness to enter ‘the cracks’.

Finally, Reddy and Amer 2022 move between a critique of the academy and radical possibilities within. The authors articulate ‘five cogs of the neoliberal machinery of the academy’ and then offer up ‘political‐personal intentions for the reorientation of the discipline of hegemonic social psychology with the aim to disrupt the politics of knowledge production and eradicate precarity’. Contesting ‘science… as a narrow patriarchal project that uses a mechanistic reductionist model created to exploit knowledge for the industrial revolution’, these writers challenge how ‘western, imperialist, whitestream academia creates and engenders precarity’ through imperial politics of knowledge production. In addition, however, they elaborate on more critical and indigenous approaches and draw expansively and generatively from the Readsura Decolonial Editorial Collective (2022a, 2022b), acknowledging the ‘collective agency (of) particular bubbles that sustain and nourish marginalized folks in the academy’. These writers call for ‘eradicat[ion of] epistemic injustice’, making space within the discipline for transformative ‘complaint’, naming our moments of collusion (in teaching and research), making visible and vulnerable the testimonios of insider–outsider researchers of colour working alongside communities under siege. They call for an investment in relationships and collectives, rather than metrics of individualism, in connections with and support for the scholars and knowledge production from the Global South. They end their submission with love and demand, ‘We present precarity as merely the lens that allows us to see the elephant in the room. Our work is not done until precarity finds no water in these new worlds and we invite you to join us, and those who have become before us, to engage in the revolutionary labour necessary to eradicate precarity in social psychology and academia’.

These three articles advance a sharp disciplinary critique–naming the harms of our discipline—but also elevate a powerful set of images of critical social psychology *otherwise*: surfacing questions about whose knowledge is centred? To whom are our projects accountable? What can be learned and kneaded in critical research collectives perched at the margins between community and academy? Can critical psychology speak at once to communities under siege and policymakers largely ignorant to the struggles on the ground? How can we interrupt the western worldview so sedimented in our disciplinary traditions, and invite a ‘view from the cracks?’

## WHAT'S LEFT TO SAY?

And so, in the sweet morning after reading and appreciating powerful interventions into critical psychology by these articles, I will pose a few loving challenges on how the authors have stirred me, and where we all—authors and readers—might wander.

### It's not just poor folks: follow the money

I so appreciate that the writers in this volume introduce material/economic precarity as a foundational element of our psychological subjectivities (rather than cordoning off the psychological as if hermetically sealed from the social/racial/political). At the same time—I want to introduce some of the thinking that Lois Weis and I have done, in an essay on *critical bifocality* (Weis & Fine, 2012), and a piece I wrote with Jessica Ruglis on *circuits of dispossession, privilege and resistance* (2008) to ask us to stretch—theoretically and epistemically—beyond the mouths that speak or the bodies that ache. Both papers call for critical psychologists to take up inquiries that centre the thick intersect of structural *and* psychological analyses, by braiding how history, structures, policies and ideologies permeate lives; that refuse downstream analyses alone; that track how power/ideologies/money/even conspiracy theories flow; that resist the temptation to see ‘the problem’ or the ‘evidence’ only in those most impacted or those most vocal.

In the case of precarity I want us to interrogate, indeed, how structural and economic precarity map onto endorsement of conspiracy theories but I also want us to lift the veil on the well‐funded billionaires and think tanks and policy groups who are pumping out these ideologies; that is, to track how these ideologies are supported by dark money elites (Bowen, 2022) committed to funding, fuelling and circulating conspiracy theories. As we write with concern about white working‐class folks mouthing and subscribing to dangerous conspiracy beliefs, it is crucial to document a fuller story, that is how the billionaire class preys on the open sores of poor and working‐class people who are searching for explanations beyond personal failure, feeding xenophobia/racism/conspiracy while occluding the tactics and grotesque profits of global capitalism and state violence. It is undoubtedly the case that low income and poorly educated folks, especially those who are white, are perhaps most susceptible to the virus of nationalism and conspiracy theorizing (by the way: is it really *conspiratorial* to think the state and global capital are out to get you?). However, social psychologists have an obligation to peer upstream to understand who is pumping money and ideas into the air we breathe, the churches we attend, the candidates who will spew and inflame hatred of immigrants, queers, people with disabilities, Muslims, BiPOC persons, women… Social psychology has an obligation to tell a thicker, more complex story of how these conspiratorial and hateful viruses enter our collective bloodstream and not reproduce the myth that only white working class carry the hate. In 2016, according to exit polls, it was America's white and wealthy voters—white college graduates and white female voters—who brought Trump to victory. As noted in *The Guardian*, ‘Far from being purely a revolt by poorer whites left behind by globalization, who did indeed turn out in greater numbers for the Republican candidate than in 2012, Trump's victory also relied on the support of the middle class, better educated and well off…. Of the one in three Americans who earn less than $50,000 a year, a majority voted for Clinton. A majority of those who earn more backed Trump’ (Henley, 2016). To engage in what we have called a circuits of dispossession analysis, one must interrogate not only who is most adversely impacted by structural inequities but also who is prospering unjustly and where do collectives of resistance gather.

### A vibrant intersectional lens on precarity

A second friendly challenge I offer to the volume concerns how we conceptualize the knotty intersections of precarity with race and class; caste, immigration, gender, sexuality and disability. Sometimes… I worry that when social scientists elevate class as a core material condition, a predictor of attitudes and behaviours, vibrant intersections are ‘whited out’. For instance, there is a curious unexplored tension in precarity studies in the United States—a spot of collective not‐knowing—about race, class, precarity and MAGA (Make America Great Again/Trump Supporters). In the United States, economic precarity clearly predicts the endorsement of MAGA ideologies, election lies, xenophobia and conspiracy beliefs in white working class and poor people in the United States (and in France and Italy). But these same empirical relationships between economic precarity and conservatism are not found, for the most part, among African Americans, Native Americans or Latinos. While these dynamics are quite complicated, the visuals of white working‐class male mobs, domestic terrorists, swarming and assaulting the US capital tell one important story, perhaps about white economic precarity. We are still left without understanding the implications of how communities of colour, poverty, sexual marginalization, engage in healing, arts, activism, protest and mutual aid—often through aesthetics and healing (see Williams, 2023)… I do not mean to romanticize (or essentialize) any ‘groups’ or ways of coping with impossible situations, but to suggest that intersectional inquiries will help us see beyond the scenes of the white mobs. How can we racialize, make more intersectional, the powerful and undeniable linkages of economic precarity and conspiracy beliefs, even as we attend to the dynamics of how race, gender, sexuality, class, disability… intersect with precarity to predict attitudes/behaviours?

### Precarity porn

Third, while I do not want to read or conduct these analyses, we must interrogate the perverse pleasures derived by those who enact or witness precarity, as state officials and everyday people dangle usually racialized/marginalized/out‐of‐place bodies across borders, separate children, lynch, threaten workers with job loss, torture the parents of trans children, watch people squirm. As frightening as it might be to examine the motives or catalogue the consequences, we need to interrogate why and also the impact on those who engage AND those who witness and are routinely exposed to such scenes. As social media captures these grotesque images of abuse, are we developing a tolerance or more frightening an addiction or taste for precarity porn? That is, how are we to understand the sadistic flows of authoritarian regimes and institutions—policing, prisons, border patrol—and the pleasures derived from enforcing/watching precarity? Dangling children at the border/threatening gay marriage/undermining reproductive justice/restricting voting rights—the sweet and sadistic smells of freedom offered and yanked away? What is precarity porn and under what conditions does it capture the popular imagination?

### Prec(ar)ious knowledge

And fourth, I enter the linguistic nesting of precious, within precarious, and wonder: When/How does precarity generate rich imaginations for art, poetry, writing, mutual aid, collectives, activist mobilizations? What is the knowledge cultivated through precarity? To what extent are these generosities and creativities inspired despite or because of?

I have been most fortunate, for the past 30 years, to work alongside movements of highly impacted people and communities, seeking justice, producing knowledge and art and theatre and science, and popular education, organizing on behalf of community‐based wisdom. For instance, most recently, during the pandemic and bold struggles for racial justice, we—a group of young high school students of colour, a middle school counsellor, an oral historian who teaches at a local community college and I—have trained the young people, as oral historians, who are gathering and archiving stories of youth and elders, growing up and old, in the precarious waters of immigration and police surveillance/potential evictions/foster care. These young people have gathered almost 100 oral histories, in a youth‐led, critical race, participatory oral history project (see Finesurrey et al., 2021 on youth‐led oral histories of living through pandemic, racial protest and online learning). In a move of epistemic justice, the precious wisdom and analyses of youth living in precarious contexts, very much on the margins, peering both out and in at a society in struggle, is quite brilliant. As an inter‐generational collective of researchers, we have presented this scholarship at the Oral History Association in Los Angeles and in London—and frankly the material is simply better—more compelling, more precarious, more valid and more policy worthy, because young people on the ground and in struggle were gathering and analysing the material. How do we speak of precious, critical knowledge cultivated in the context of communities under siege, without either romanticizing the youth researchers or whiting out the oppression of everyday life?

### Critical psychology and movements: knowledge generated in entangled struggles and solidarity

Finally, as a vision of what critical psychology might be otherwise or put differently what is the obligation of university researchers, I want to lift up rich examples of what we have been calling solidarity studies. At the Public Science Project in New York City, and the Inside/Outside Prison project at the University of South Africa, I have had the joy/opportunity to collaborate with women in prison and just out of prison, living in the hell of incarceration and the deeply troubling fog of ‘freedom’. In New York, we are a research collective of women still inside, many outside, two lawyers and two researchers. We have been documenting and organizing around the ‘doubled violence’ that links domestic violence and the criminalization of survivors, particularly women and femmes, particularly Black and Latina (see Survivors Based Justice Project, 2021). With similar commitments to movement‐led anti‐racist research, Brett Stoudt, Brittany Moreira and the collective Communities for Police Reform (https://morrisjustice.org/about/), have mobilized a city‐wide research collective rooted in the community demands for divesting in policing, abolition of prisons, investing in mental health/restorative justice, and building safety in communities of colour. Led by residents and activists from communities of colour that endure massive levels of racialized police violence (physical, emotional and sexual), the Black Law Project, Communities for Police Reform and Raise the Age projects are rooted in knowledge production/organizing and policy reformation, situated in struggle. A third example comes from Jennifer Ayala et al. (2022) at Saint Peter's University at The Center for Undocumented Students, where they have launched a series of activist/healing/research projects led by families under immigration scrutiny/assault/surveillance, families and students seeking sanctuary and justice. And a fourth example comes from Monique Guishard and her colleagues at the Bronx CRIB (community‐based IRB ‐ http://bxcrrb.org/team‐showcase/dr‐monique‐a‐guishard/) where community members review and critique, challenge and hold accountable grants submitted to Einstein Hospital asking about who gets the money? What language will the researchers use to describe participants? Are you only studying diabetes, or will studies of schizophrenia or STDs suddenly pop up in your work?

All of these projects are rooted in epistemic justice, anti‐racist solidarity and struggle, curated to accompany movements for justice, designed to challenge dominant lies and to tell another story about what might be. Critical participatory action research may be a radical tactic for honouring and centring precious and brilliant knowledge cultivated in conditions of oppression, sharpened in struggle and focused on root causes, consequences and radical alternatives to precarity (Fine & Torre, 2021).

As Gramsci commented almost a 100 years ago, we find ourselves in yet another, or a continual interregnum. The morbid symptoms remain, multiply and are tweeted now. The deeply embodied and stratified consequences of precarity on full display, even as epistemologies of ignorance, (Mills, 1999) refusal, silencing and necropolitics (Mbembe, 2019) saturate our global consciousness, insinuated within academic scholarship.

A century later, we confront again our responsibility as public intellectuals—to name, honour, refuse to narrow the aperture, critique our own academic institutional collusion, work in fierce and soft solidarity with activists and scholars in our communities and most particularly in the Global South. We labour to challenge the western worldview, the white gaze, understand precarity as a global and strategically targeted assault that is, indeed, colonial, historic, cumulative, cascading, criminalized, structural, psychological, maddening and sometimes a source of brilliance, care, humour, joy and good trouble. We write with/beside/for movements for justice… still thinking and growing a work that must be done with/not on, communities under siege, fuelling prec(ar)ious freedom dreams.

## CONFLICT OF INTEREST

All authors declare no conflict of interest.
